# Postgraduate health promotion students’ perceptions of at-risk populations: Those who smoke tobacco, are overweight or obese or drink alcohol at hazardous levels

**DOI:** 10.1371/journal.pone.0241076

**Published:** 2020-10-22

**Authors:** Marguerite C. Sendall, Alison Brodie

**Affiliations:** 1 School of Public Health and Social Work, Faculty of Health, Queensland University of Technology, Kelvin Grove, Queensland, Australia; 2 Institute of Health and Biomedical Innovation, Queensland University of Technology, Kelvin Grove, Queensland, Australia; University of Turin, ITALY

## Abstract

Perceptions acquired during tertiary health promotion education can influence students’ interactions with their future service-users. Reflective practice can highlight these perceptions. Here we describe the findings of a reflective exercise conducted with postgraduate health promotion students as part of a learning activity. Students (n = 44) reflected on their understandings of at-risk populations in three priority areas—tobacco, obesity and alcohol. The activity aimed to deconstruct students’ understandings of these populations and identify understandings juxtaposed to the philosophical underpinnings of health promotion, for addressing through teaching and learning. Thematic analysis revealed students’ understanding of all three at-risk populations fit within five themes: apathy/lack of altruism, complexity/choice, pressure/control, escaping /excuses, and environmental contexts. Students also have varying levels of tolerance to at-risk populations, expressing greatest tolerance towards those whose addiction undermines choice and self-control, and least towards those who are overweight/obese or whose behaviour causes risk to others. Our findings show reflective practice is a valuable tool to help educators understand students’ attitudes and values and implement changes to support their future role in the community.

## Introduction

The education of health promotion practitioners plays a key role in developing a resilient workforce. However, health promotion is a complex field of study because it addresses multifaceted problems, involves a range of methods and theories [1] and encounters ‘situations of complexity and uncertainty’ [2]. Each new generation of health promotion students introduces new issues to consider. This causes those who teach health to reflect on pedagogical methods.

Perceptions held or acquired during tertiary study can influence health promotion students’ interactions with their future clientele and their long-term sustainability as health promotion practitioners [3]. For example, the professional need to act on health issues from a medical perspective sometimes hinders a more reflective, analytical, or empathetic attitude towards the challenges faced by clients [1]. For this reason, it is important educators create a learning environment aligning with the core health promotion principles of social justice and equity, especially in the final years of study. This enables health promotion students to develop and maintain a positive and empathetic attitude, especially for those populations who are at-risk and/or marginalised [4].

Reflective practice is now a respected learning tool in many medical and allied health programmes to help practitioners align their personal views with the fundamental guiding principles of their professional practice [5]. Learning reflections may uncover personal insights affecting the real-world application of health promotion skills and knowledge. These can be addressed in pedagogical discussions. There is a considerable body of literature addressing the value of reflective practice for student learning [6, 7]. Good reflective practice can enhance critical thinking and professional capabilities such as empathy which is important for health promotion practitioners [4, 8]. Creating opportunities for self-reflection is becoming increasingly important for health promotion courses [6, 9], however, there is little literature describing the use of reflective practice in public health education [9], especially in the later years of study.

The motivation to create a resilient and empathetic workforce inspired the first author—a tertiary health promotion educator—to undertake a qualitative learning activity (a reflective exercise) with postgraduate health promotion students, to deconstruct their understandings of at-risk populations in three health priority areas—overweight/obesity, smoking tobacco, and drinking alcohol at dangerous levels. The primary purpose of this activity was for quality assurance—to identify areas of misalignment with core health promotion principles such as social justice and equity, for addressing through teaching and learning. This article describes the findings of this learning activity and makes a significant and novel contribution to the field of health promotion pedagogy.

## Materials and method

### Respondents

All respondents are postgraduate public health students at a large Australian metropolitan university. Respondents were enrolled in Contemporary Health Promotion (2012, 2013 and 2014) which is a foundational and elective unit in the health promotion major of the Master of Public Health. Students were enrolled in eight postgraduate courses from health science, public health, psychology and study abroad. Refer to Table 1: Respondents course enrolment.

**Table 1:** Respondents course enrolment.

Course Enrolment	*HL30 GC Chronic Health Conditions*	*HL38 GC Health Science*	*HL68 GC Health Science*	*HL88 M Health Science*	*PU60 GD Public Health*	*PU85 M Public Health*	*PY41 GD Road Safety*	*UO80 University Study Abroad Certificate*	Total
2012	0	9	1	1	3	1	0	0	15
2013	0	2	1	5	1	5	0	1	15
2014	1	0	0	2	2	6	1	0	12
Total	1	11	2	8	6	12	1	1	42

The total enrolment across all three years was 42 students. Of these students, 38% (n = 16) were enrolled in the unit internally and 62% (n = 26) were enrolled in the unit externally. For course enrolment, 38% (n = 16) of students were enrolled internally, 57% (n = 24) were enrolled externally and 5% (n = 2) were enrolled in multi-modal delivery. According to attendance type, 69% (n = 29) were part-time students and 31% (n = 13) were fulltime. Eighty-three percent (n = 83) of students were domestic and 17% (n = 7) of students were international.

### Data collection

The learning activity consisted of three open-ended questions posted on the unit’s e-learning site: *What do you think about people who smoke tobacco*? *What do you think about people who are overweight or obese*? *What do you think about people who drink alcohol at hazardous levels*? Completion of the learning activity was voluntary. The learning activity was implemented in the first three weeks of semester across three consecutive years. In the first year, 13 respondents completed the questionnaire, while 18 and 13 respondents completed the questionnaire in the following two years, respectively. The total response rate was 100%. The discrepancy between student enrolments and respondents is explained by 2 late unit enrolments in 2012, 3 unit withdrawals in 2013 and 2 late unit enrolments in 2014.

Completed and de-identified responses were returned by email, downloaded, and all respondents were identified with a logical code. The respondents’ original emails were deleted. All respondents answered all questions, however some respondents expressed difficulty in identifying specific issues within each question, suggesting all risk factors were underpinned by the same issues.

### Data analysis

All questionnaires were electronically formatted for analysis and printed in hard copy. Data analysis was inductive to ensure respondents’ voices were heard. The first author holds a PhD in health promotion and is an experienced tertiary educator with high teaching scores. The author bracketed preconceived ideas and assumptions during the data analysis phase to minimise bias. There were six stages in the analysis process.

The first stage of inductive data analysis was reading. All transcripts were read for initial impressions.

The second stage focused on personal understanding identified by ‘I’ and ‘me’ statements to highlight contextualised significant statements. Significant statements were identified across questions rather than per transcript. For example, significant statements were highlighted in the responses to the question about tobacco smoking for all years, before highlighting significant statements in the responses for overweight and obesity for all years. Each question was assigned a highlighter colour. Statements which reflected an academic view were not highlighted. The third stage was coding. Significant statements were allocated an open code to broadly represent the understanding. Then each significant statement was coded within the open code. This process was repeated several times until all the significant statements were coded to represent a specific understanding. The fourth stage was grouping. Codes were grouped within each question—tobacco, overweight and obesity, and alcohol. Significant statements were assessed for internal homogeneity to ensure all codes were in the correct group. This was done by comparing and contrasting each code in each group. Some codes were moved to other groups. Some codes were removed from groups. The final groupings within the tobacco question were addiction, coping/stress, choice/right, and image. The fifth stage was theming. Final groups were assessed for external heterogeneity so there was a clear albeit related distinction between each theme. The sixth stage was labelling. Quotes from the text were identified as labels to represent the wholeness of each theme.

### Ethics

The primary purpose of this activity was quality assurance. Queensland University of Technology UHREC exempt this study from human ethics review and approval according to the National Statement on Ethical Conduct in Human Research 2007. Consent for publication was not required because this activity was exempt from ethics approval. The activity was conducted ethically and in accordance with the Ethical Considerations in Quality Assurance and Evaluation Activities [10].

## Results

Analysis reveals five (5) themes which represent postgraduate health promotion students’ perceptions about at-risk populations.

### Theme 1: Respondents’ understandings of at-risk populations as apathetic and lacking altruism

In this theme, respondents speak about the at-risk populations as being apathetic, selfish and lacking altruism. Respondents indicate this is because people who smoke tobacco lack a sense of altruism because they are addicted to nicotine, de-sensitised to health risk messages and do not care about others exposed to their second-hand tobacco smoke. Respondents intonate people who are overweight or obese are apathetic because they believe overweight and obese sizes are seen as ‘normal’ and do not understand the risk of chronic disease. Respondents speak about people who drink at hazardous levels as selfish because they do not understand the extent to which their behaviour might hurt themselves or impact on others.

In this theme, respondents convey a range of emotive responses about these at-risk populations. These emotions include feeling sorry, feeling annoyed, respect, apathy, altruism and understanding addiction.

Respondents describe two significant reactions to people who smoke tobacco, are overweight or obese, or drink alcohol at hazardous levels. Respondents feel sorry for those who smoke tobacco and have unsuccessfully tried to quit. Equally, they feel sorry for those who try and do the right thing by eating healthily but find it difficult to manage their weight. Respondents feel sorry for those who are overweight or obese because they have ‘normalised’ their size and may not understand the risks associated with being overweight or obese.

“It depends on the other aspects of their lifestyle and how obese they are. For example, if they are living a healthy lifestyle with correct diet and exercise regimes and are still overweight or obese I often feel sorry for them, because they are doing the right things but still not getting results.”

**S120125SL**

Respondents feel annoyed by those who smoke tobacco and drink alcohol at hazardous levels because they are seen as putting themselves at risk of heart disease and other conditions. Respondents are annoyed others, such as young families, are put at risk of inhaling second-hand tobacco smoke and exposed to the negative effects of drinking alcohol at hazardous levels. Respondents are annoyed they have chosen not to smoke tobacco but are exposed to second-hand tobacco smoke. Respondents are annoyed by those who smoke tobacco because they hide behind the excuse of addiction, and by those who are overweight or obese people because they have made habit of being lazy.

“People who are overweight or obese because they are too lazy, annoy me, especially if they have a young family because the development of a chronic disease or premature death can affect their loved ones significantly.”

**S120125SL**

“I also get annoyed at people who smoke at work as they have more ‘breaks’ than other staff members.”

**S120125SL**

Respondents feel most people who smoke tobacco respect others however there is a subgroup of these people who lack respect for others. Respondents indicate this is seen through a range of behaviours and actions, for example, second-hand tobacco smoke, smoky environments, flicking butts on the ground and taking more breaks at work than non-smokers.

“I think it is a personal choice individuals make, however it sometimes annoys me when individuals smoke near or around me. I feel they are choosing to smoke, and I am choosing not to smoke so they should be respectful not to smoke near me.”

**S1201312MS**

Respondents feel sorry for these people because they have become apathetic to addressing the risk factor and reflect a level of selfishness. This is seen as a lack of altruism, or not having the intellect to think about and care for themselves and others.

“I think people who smoke tobacco do not care as much as about their physical health, long term, in comparison with smoke-free individuals. People who smoke also have a disregard for the people they live with, as secondhand smoke can also influence their lives as well.”

**S120125SL**

Respondents exhibit understanding for those who smoke tobacco because it is related to nicotine addiction and other life stressors. Respondents feel nicotine is a powerful addiction but do not understand why this group of people continue to smoke tobacco. Similarly, respondents show understanding towards those who drink alcohol at hazardous levels because they see a separation between the person and the behaviour.

“Smoking is addictive and, like any addiction, is often ignited by outside influences and maintained by a personal inability to quit. Smoking is a part of society, and I believe, always will be. Smokers are merely another group of the population addicted to a substance for whom we need to provide ongoing education and support in order to, hopefully, help them overcome the addiction.”

**S120136FrF**

This respondent highlights the complexity of nicotine addiction and stress by telling a story about friends who are pregnant. The respondent knows the friends are aware of the health consequences for themselves and their unborn baby and feels they are unable to quit because they are addicted to nicotine.

“I also see how addictive behaviors and stress can lead to issues of quitting. My two closest friends smoke and while I care for their health and know that they are fully aware of the consequences to not only their own health but that of their children they still continue to smoke. This had actually become an unspoken tension between myself and one of my friends as he is now pregnant and not quitting but cutting down from a pack a day to 10 (which is still way to much) I have tried to breach the subject with her but beyond “are you sure you should be doing that?” I feel powerless to help her as she smokes to cope.”

**S120122DT**

### Theme 2: Respondents’ understandings of at-risk populations as complex and making choices

In this theme, respondents speak about the at-risk populations as complex and making choices. Respondents say this is because people who smoke tobacco experiment without understanding the consequences, and ultimately, it’s a choice. Respondents state people who are overweight or obese cannot claim ignorance but their choices are complicated by living in complex obesogenic environments. Respondents indicate people who drink at hazardous levels make socially desirable and accepted choices.

In this theme, respondents suggest ardent responses about these at-risk populations, which include ideas related to choice, responsibility, power and control and complexity. Respondents feel choice is an absolute concept available to those who smoke tobacco and drink alcohol at hazardous levels but is not absolute for those who are overweight or obese. This is seen within the context of addiction.

“No one gets fat overnight. It is the result of a person’s choices about what they put in their mouth. While losing and maintaining weight requires a conscious effort for most, it seems a majority of these people are not taking responsibility for their own health.”

**S1201318JrJ**

Respondents view people in at-risk groups as irresponsible and not taking care of themselves or others. Respondents suggest people in these groups chose the path of least resistance because it is easier to not address the risk factor—for example, not to lose weight or give up smoking tobacco. Respondents infer these people should be more accountable to themselves, their families and the broader community.

“From my experience with obese and overweight people, I think they are divided into two categories. First one is when they make themselves obese by making bad or unhealthy food choices. Additionally, they chose to be physically inactive. These kind of overweight or obese individuals are responsible somehow for their condition. On the other hand, the other kind of these individuals might be affected by some predisposed factors that led them to be obese or overweight, such as medical conditions or some medications that affect their weight.”

**S120141QSB**

Respondents suggest people have the power and control in their lives to make healthy choices despite the social and physical contexts. That is, everyone can be empowered and take control of their lives.

“Drinking alcohol is another thing than smoking and being overweight. Drinking alcohol can hurt other people, not just you. Therefore I don’t accept it when people drink so much that they can’t control themselves and might hurt somebody.”

**S1201314ST**

Respondents view people in these groups as complex and multidimensional but suggest there needs to be a tougher, more direct approach with affirmative action to deal with these complexities.

“I think that at face value, as a non-smoker it is easy to say smokers should ‘just give up’ but it is much more complex than that, it is a proven addictive substance and behaviour therefore it is more than just a matter of self-control”.

**S120124RK**

### Theme 3: Respondents’ understandings of at-risk populations as pressured and being controlled

In this theme, respondents speak about the at-risk population as pressured and being controlled, because people who smoke tobacco are pressured by their social environments despite the pressure of anti-tobacco legislation. Respondents indicate people who are overweight or obese are controlled by entrenched habits and a poor quality of life. Respondents intonate people who drink at hazardous levels are compromised by social pressure and norms and are powerless to step aside from excessive drinking despite health promotion messaging.

In this theme, respondents present a limited range of responses about these at-risk populations. These emotions include acceptance, frustration and contextual influences.

Respondents intonate a view of acceptance towards those who smoke tobacco because smoking tobacco is still socially acceptable in some contexts. Respondents suggest a level of acceptance towards those who drink alcohol at hazardous levels because of the associated concept of peer pressure. However, conversely, respondents indicate a lack of acceptance of those who drink alcohol at hazardous levels because of a loss of control of their behaviour and the risk of harming others.

*“In addition to that*, *tougher laws need to be created such as the plain packaging laws which will denormalise the smoking behaviour to decrease smoking uptake*. *Whilst a continued increase in the cost of cigarettes will increase quit attempts*. *Tobacco smoking is the most preventable cause of morbidity and mortality today*, *therefore changes must be made*.”

**S120124RK**

Respondents are frustrated by people in these groups because they feel powerless to help them.

“Most of the times the alcohol drinkers tend to think that drinking will provide a relief from existing problems. However, when they get sober again, they are normally affected by a hangover, which might require one or more bottles of alcohol to get over. In addition, the problems that they were shunning away from are always there when they get sober. Such scenario renders them to drink more and it becomes habitual.”

**S1201316KT**

Respondents indicate the external environmental and social influences continue to exert a level of influence on people in these groups due to peer pressure and a lack of control over their lives.

“In other cases, peer pressure especially amongst young people might contribute to hazardous drinking. Most young people think that it is ‘cool’ or ‘modern’ to drink alcohol and that everyone else drinks. The most dangerous part of hazardous drinking is that most drunkards lose sense of control hence they are prone to act irresponsibly. This renders them dangerous to themselves and to the community.”

**S1201316KT**

### Theme 4: Respondents’ understandings of at-risk populations as escapists and using excuses

In this theme, respondents speak about the at-risk populations as being escapists and using excuses. Respondents state this is because people who smoke tobacco may have mental health concerns and smoking tobacco offers some respite. Respondents indicate people who are overweight or obese deny the health risks by offering a range of excuses to justify their health status. Respondents say people who drink at hazardous levels self-medicate to escape troubles but being drunk is not an excuse for their behaviour.

Respondents expressed frustration towards people in these groups because they are seen as avoiding their problems and using alcohol as a medication. Respondents felt they were drinking alcohol at hazardous levels to mask other serious problems such as mental health issues.

*“I think some people use it as a masking agents for other issues e*.*g*. *PTSD or depression*. *Strangely my views on people who drink at hazardous levels aren’t as bad as people who smoke*, *unless they are putting other people in danger e*.*g*. *drink-driving*. *Hazardous levels of alcohol may not happen every day like smoking*, *however for some people it is an addiction and disease and needs to be treated as such*.”

**S120125SL**

### Theme 5: Respondents’ understandings of at-risk populations as recipients of environmental contexts

In this theme, respondents speak about the at-risk populations as recipients of environmental contexts. Respondents intonate this is because people who smoke tobacco may be from marginalised groups or poor socio-economic backgrounds who have little power to modify their environments. Respondents indicate people who are overweight or obese may live in neighbourhoods which have low walkability and a high density of fast food outlets. Respondents state people who drink at hazardous levels live in social and geographic environments which enable drinking at hazardous levels.

In this theme, respondents convey sensitive responses about the impact of the social and environmental contexts in which these at-risk populations live and work.

Respondents state the social and cultural environment, and the social determinants of health play a big role in the risk factors of people in these groups. Respondents understand the role of the social and physical environments and the impact they have on people in these groups.

“Where I live it is very easy for people to make bad food choices as there are so many unhealthy fast food options and very limited healthy fast food options. Again my opinion is not on the people but the environments that make the unhealthy options easier than the healthy options.”

**S120126ET**

## Discussion

Postgraduate health promotion students express a variety of understandings about populations at risk through overweight/obesity, smoking tobacco or consuming alcohol at dangerous levels. Similar to the findings of others [11], our postgraduate cohort express a general understanding of the complex, multifactorial nature of health determinants and an awareness of the impact of context and environment in at-risk populations. However, students show variable tolerance to these at-risk populations, ranging from high tolerance (expressed as empathy and understanding), to low tolerance (expressed as annoyance, anger and frustration).

A higher tolerance is expressed for those whose addictions undermine choice and self-control, whose environment makes choosing unhealthy options easier than choosing healthy ones, and whose risky behaviour aligns with social norms. Here, students’ lived experience may influence their level of tolerance. The healthcare workforce is known to exhibit similar health risk behaviours to the general population [12], and it has been shown bodyweight, tobacco use, and alcohol consumption in health promotion students [13] and other health professionals [14–16], influence their tolerance of these practices in others. Students’ personal experience with health risk behaviours was not explored in this learning activity. A more structured reflection would be useful to explore this in future studies.

Postgraduate health promotion students express low tolerance towards those whose behaviour causes risk to others, such as through alcohol-fuelled violence or by smoking while pregnant or in close proximity to others, especially children. Many students are also intolerant of those who are overweight or obese. This aligns with findings from other studies showing a greater bias against ‘fat’ people than other commonly stigmatised groups such as smokers and heavy drinkers [17]. Anti-fat bias has been shown to be as strong or stronger in health professionals (including doctors, nurses, dieticians and exercise scientists) than in the general population [17].

While there has been little research about bias or prejudice in health promotion students, studies in medical and nursing students [5] showed many express biases towards their patients, which they believed was likely to influence their future practice. Students’ professional experience may impact on these attitudes. Postgraduate health promotion students are likely to come from graduate studies, perhaps even a professional background, in a health or allied field, giving them a greater breadth of knowledge and experience than undergraduate students. However, research shows empathy tends to decrease in students as healthcare education progresses [18], possibly because of the pervasiveness of the biomedical model and the focus on physiology and pathology at the expense of ‘softer’ skills such as empathy [19]. That some of our cohort articulated a lack of tolerance and empathy, suggests curriculum should focus on facilitating greater understanding of the complex determinants contributing to risk, especially in relation to overweight/obesity.

Tertiary courses specifically focussed on students’ biases or negative attitudes identified in reflective practice can help reduce those biases [20]. For example, Rote et al. [21] showed a college course focused on reducing weight bias facilitated significant reductions in this bias among health promotion undergraduates, with students showing “more nuanced understandings” of obesity post-semester. Further, Kempenaar and Shanmugan [19] suggest face-to-face meetings with ‘third sector’ organisations and service-users help transform students’ understanding of the users’ circumstances, history, culture and experiences. This experience is most valuable early in a course when students are purposively developing attitudes and behaviours to take into their professional lives.

In teaching and learning to address overweight/obesity, health promotion educators should give equal relevance to the genetic, social, and environmental causes of overweight/obesity as to the traditional information on causes and treatments and should model non-judgemental language and behaviour [17]. Educators are role models for their students, and it is prudent they also reflect on their own health beliefs and ask whether these align with their actions and advocated ethical stance. Attitudes and dispositions such as empathy and tolerance are crucial to health promotion practice but are much less able to be taught didactically [22]. Here, more structured reflective exercises may facilitate students’ exploration of the complexities of health risk behaviours within a broad socio-ecological context [22] and with a greater understanding of the lived experience of others.

## Strengths and limitations

Several factors may limit the generalisability of our findings. Postgraduate health promotion students with a background in nutrition and dietetics may bias responses against those who are overweight and obese. There was no information on students’ demographic characteristics or personal behaviour with respect to smoking tobacco, overweight/obesity and hazardous use of alcohol—factors which may have influenced their responses. Additionally, there was a poor response to the learning activity at the end of the course. As a result, it was not possible to determine a) if students changed their understandings after completing the course, or b) if students found the opportunity for self-reflection useful for their professional development. Greater insights would be achieved by repeating the learning activity in additional units and throughout postgraduate students’ tertiary education, to determine how themes emerge or change with time and further learnings.

## Conclusion

This reflective learning activity showed postgraduate health promotion students have varying perceptions of, and levels of tolerance to, at-risk populations. Our findings suggest curriculum should focus on facilitating greater understanding of the complex determinants contributing to overweight/obesity, smoking tobacco and drinking alcohol at dangerous levels, and should consider genetic, social, and environmental factors. Further research is required to understand how more regular and structured reflections involving student’s prior personal and professional experience can address bias and help develop empathy and tolerance.
